# 40 Years of Percutaneous Coronary Intervention: History and Future Directions

**DOI:** 10.3390/jpm8040033

**Published:** 2018-10-01

**Authors:** John Canfield, Hana Totary-Jain

**Affiliations:** Department of Molecular Pharmacology and Physiology, Morsani College of Medicine, University of South Florida, 12901 Bruce B. Downs Blvd, MDC08, 2170, Tampa, FL 33612, USA; jcanfie1@health.usf.edu

**Keywords:** coronary artery disease, angioplasty, percutaneous intervention, stent, restenosis, reendothelialization

## Abstract

The field of interventional cardiology has evolved significantly since the first percutaneous transluminal coronary angioplasty was performed 40 years ago. This evolution began with a balloon catheter mounted on a fixed wire and has progressed into bare-metal stents (BMS), first-generation drug-eluting stents (DES), second- and third-generation biodegradable polymer-based DES, and culminates with the advent of bioabsorbable stents, which are currently under development. Each step in technological advancement has improved outcomes, while new persisting challenges arise, caused by the stent scaffolds, the polymers employed, and the non-selective cytostatic and cytotoxic drugs eluted from the stents. Despite the promising technological advances made in stent technology, managing the balance between reductions in target lesion revascularization, stent thrombosis, and bleeding remain highly complex issues. This review summarizes the evolution of percutaneous coronary intervention with a focus on vascular dysfunction triggered by the non-selective drugs eluted from various stents. It also provides an overview of the mechanism of action of the drugs currently used in DES. We also discuss the efforts made in developing novel cell-selective drugs capable of inhibiting vascular smooth muscle cell (VSMC) proliferation, migration, and infiltration of inflammatory cells while allowing for complete reendothelialization. Lastly, in the era of precision medicine, considerations of patients’ genetic variance associated with myocardial infarction and in-stent restenosis are discussed. The combination of personalized medicine and improved stent platform with cell-selective drugs has the potential to solve the remaining challenges and improve the care of coronary artery disease patients.

## 1. Introduction

**Figure 1:**
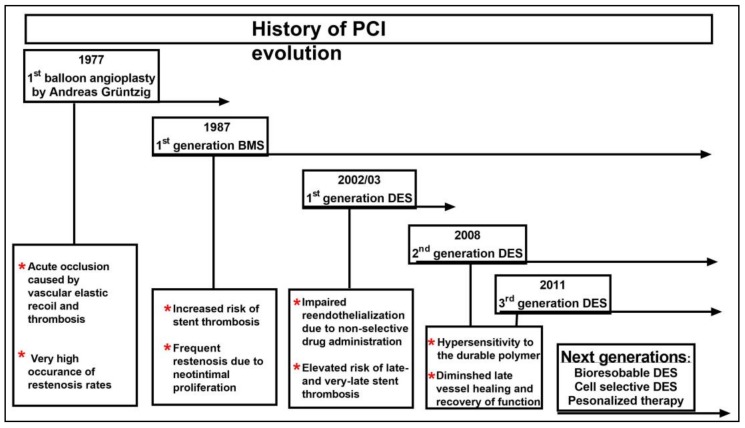
Evolutionary history of percutaneous coronary intervention (PCI). ^1^ BMS: Bare-metal stent. ^2^ DES: Drug-eluting stent.

Worldwide, coronary artery disease (CAD) is the leading cause of morbidity and mortality and imposes a major health and economic burden on the majority of developed nations. In the United States alone it is estimated to cause 790,000 heart attacks each year [1] with an estimated cost of $89 billion as of 2016 that is expected to increase to $215 billion by 2035 [2]. In the past decade, improved therapies have decreased the mortality accompanying CAD while increasing survival following a myocardial infarction. Despite this decrease in mortality, the prevalence of CAD is expected to continue to increase due to the increase in the aging population [3]. The treatment of CAD has been transformed by the introduction of percutaneous coronary intervention (PCI), which remains the focus of intensive research and development (Figure 1) [4,5]. 

The first milestone in treating CAD was achieved by the introduction of Balloon angioplasty performed by Andreas Grüntzig in 1977. However, this technique had two major drawbacks: Thrombosis and acute occlusion caused by vascular elastic recoil occurred in 5–10% of patients immediately after the procedure, and development of neointimal proliferation with restenosis occurred in ~30% of patients within the first six months. In an effort to combat the shortcomings of elastic recoil, pioneering work performed by Sigwart et al. developed and implanted the first self-expanding bare-metal stent (BMS) following balloon angioplasty, and in 1987 the BMS was the first food and drug administration (FDA)-approved stent in the USA [6]. Although this new technology reduced early elastic recoil, it was accompanied by two major problems: stent thrombosis and in-stent restenosis (ISR) [7]. Despite the potentially serious complications associated with the procedure, BMS implantation became the standard of care following the publication of the results from two landmark trials in 1993, the STRESS and the BENESTENT, which indicated that BMS implantations were superior to balloon angioplasty alone [8]. However, follow-up studies found that in-stent restenosis due to neointimal proliferation was still persistent at the rate of 20–30% [9]. 

## 2. Vascular Response to Stent Deployment

During the 1990s, extensive investigations sought to elucidate the molecular mechanisms underlying the vascular response to PCI and stenting. The actual use of balloon angioplasty together with stent deployment disrupts the endothelial cell (EC) layer and initiates a cascade of molecular events that contribute to thrombosis and restenosis. This inevitable EC injury induces platelet activation and aggregation followed by infiltration of leukocytes and monocytes into the lesion site. The resulting inflammatory response plays a critical role in the initiation and progression of neointimal formation. Platelet and inflammatory cells secrete growth factors, chemokines and cytokines, and induce macrophage phagocytosis that clears cell debris and induces proliferation and migration of quiescent vascular smooth muscle cells (VSMCs) and ECs to heal the lesion. The shift in VSMC phenotype from quiescent contractile to synthetic, and subsequent entry into the cell cycle followed by migration into the intima and deposition of the extracellular matrix is the hallmark of intimal hyperplasia. 

## 3. The Evolution of the First Generation of Drug-Eluting Stent

Several therapeutic approaches aimed at reducing neointimal hyperplasia have been explored. Although targeting platelet activation, thrombosis, or inflammation did not confer any benefit, inhibition of VSMC proliferation and migration in response to antiproliferative drugs such as sirolimus and paclitaxel, substantially reduced intimal hyperplasia in animal models when given systemically or when coated on the surface of bare-metal stents. 

Sirolimus, also known as rapamycin, is a natural macrocyclic lactone obtained from the bacterium *Streptomyces hygroscopicus* found in soil samples from Easter Island (Rapa Nui) [10] that is a potent antifungal, immunosuppressive and antiproliferative agent. Its lipophilic properties enables sirolimus to pass through cell membranes and then bind initially to its intracellular receptor FKBP12 and consequently to the mammalian target of rapamycin complex 1 (mTORC1) resulting in inhibition of its serine/threonine kinase activity. mTORC1 is a multiprotein complex that regulates cell proliferation by controlling the levels of cyclins and cyclin-dependent kinase (CDK) inhibitors required for G1 to S cell cycle stage transition. By inhibiting mTORC1, sirolimus blocks the action of mitogenic stimuli to downregulate the CDK inhibitor p27^Kip1^ (p27) and ultimately inhibit both cyclin E-CDK2 and cyclin D-CDK4 complexes. The resulting increase in p27 levels is the final pathway by which sirolimus exerts its antiproliferative effects. Paclitaxel is a natural compound isolated from the bark of the Pacific yew tree and is a potent cytotoxic drug. Its lipophilic properties enable paclitaxel to pass freely through the cellular membranes and then promote microtubule assembly, which leads to arrest of the cell cycle during the G2/M-phase and eventual apoptosis [11,12,13].

In 1999, Edwardo Sousa implanted the first sirolimus-eluting stent (SES). Several randomized controlled trials that followed (RAVEL, SIRIUS, E-SIRIUS, C-SIRIUS and ISAR-DESIRE) revealed that SES was superior to BMS in reducing ISR and target lesion revascularizations [14,15,16,17]. In 2003, the FDA approved the SES, CYPHER, and shortly after the paclitaxel-eluting stent (PES), TAXUS. However, follow-up studies showed that patients receiving drug-eluting stent (DES) were at higher risk of developing late clinical events such as myocardial infarction and death owing to late stent thrombosis (ST), when compared to BMS [18,19]. This devastating complication imposed the use of prolonged regimens of dual anti-platelet therapy [20,21,22]. 

## 4. Vascular Response to Drug-Eluting Stent

The increased incidence of DES-associated late ST has been attributed primarily to the lack of reendothelialization of vessel walls with competent ECs. A competent endothelium (both in integrity and function) is critical in order to provide an efficient semipermeable barrier capable of regulating vascular tone, lipid, and tissue-fluid homeostasis, as well as suppressing intimal hyperplasia, inflammation, and thrombus formation. However, DES deployment inevitably disturbs the normal competent endothelium structure. Compounding this, elution of non-selective cytostatic or cytotoxic drugs drastically reduces the quality of vessel healing and the regenerating endothelium. The exposure of the metal struts of the stents to the circulation results in hypersensitivity reactions, platelet adhesion, and chronic inflammation. Moreover, accelerated neoatherosclerosis in the stented segment, caused by the poorly formed endothelial cell junctions and impaired barrier function that allows lipoproteins to enter the sub-endothelial space, were found to occur more frequently and at an earlier time point in DES when compared with BMS [23].

## 5. New Generations of Drug-Eluting Stent

To combat the safety concerns related to incidence of ST, second-generation DES were developed. Improved platforms, made of cobalt–chromium (CoCr) or platinum–chromium (PtCr), reduced thickness and were used to improve radial strength and visibility, while newer derivatives of sirolimus, such as everolimus and zotarolimus, were used to improved lipophilicity and enhance cellular uptake. 

Second-generation DES showed superiority to first-generation DES, not only with lower target lesion revascularization rates, but also lower rates of ST with no major difference among cobalt-chromium-everolimus eluting stent (CoCr-EES), cobalt-chromium-zotarolimus eluting stent (CoCr-ZES) or platinum-chromium-everolimus eluting stent (PtCr-EES), according to large randomized controlled trails enrolling thousands of patients [24,25,26,27,28].

To overcome the hypersensitivity reaction to the durable polymer, non-polymeric third-generation DES with biodegradable polymers and a semisynthetic analogue of sirolimus, biolimus A-9, with 10 times higher hydrophilicity were also developed. These biodegradable polymer-based DES showed similar safety and efficacy outcomes to the second-generation DES and received FDA approval in 2015. 

In parallel, fourth-generation DES constructed with fully bioresorbable scaffolds (BRS), designed to provide vessel support and deliver the antiproliferative drug to prevent neointimal proliferation for a defined period after PCI, followed by gradual resorption leaving behind no permanent foreign material. Considering stent thrombosis typically forms due to impaired reendothelialization of the metallic stent, BRS appeared to be a promising candidate to improve vessel healing after PCI. The most studied bioresorbable Vascular Scaffold system was the Absorb GT1, made of fully biodegradable poly-l-lactic acid that controls the release of everolimus. Initial short- and long-term follow-up studies demonstrated the feasibility of using BRS and indicated low rates of major adverse cardiac events [29,30,31]. In 2016, the FDA approved Absorb GT1 for use in the United States. However, a larger-scale trial with long-term patient follow-up comparing the Absorb BRS to a CoCr-EES indicated an increased risk for scaffold thrombosis in the BRS group [32]. Additional studies of the Absorb BRS have also indicated an increased risk of myocardial infarction and scaffold thrombosis in patients implanted with the Absorb BRS [33]. In 2017, the FDA released a warning related to the increased incidence of device thrombosis and it was subsequently removed from the global market. 

## 6. Future Directions for Percutaneous Coronary Intervention

### 6.1. Selective Anti-Restenotic and Prohealing Drug

Despite an impressive list of promising technological advances accompanied by serious research efforts expended in the field of stent therapy over the past two decades, restenosis and ST (primarily late and very late) remain the principal factors contributing to stent-associated morbidity and mortality rates. First-generation DES effectively suppressed neointimal growth, but at the expense of poor strut coverage with incompetent endothelium [20,34,35,36,37,38]. Newer generations of DES, such as ZES achieve greater strut coverage with their low-dosage, rapid drug release, but at the expense of greater neointimal growth [39]. These detrimental trade-offs in DES technology remain unresolved, and the inability to deliver cell-selective therapy has hindered progress in the field of percutaneous intervention. 

Newer generations of DES, with more sophisticated scaffolds, thinner struts, biodegradable polymers, and even BRS, have been developed to combat the incidence of ST. Nevertheless, they still deploy the same non-selective drugs (paclitaxel or sirolimus or its analogs, everolimus, zotarolimus and biolimus, with improved lipophilicity) [40]. Current therapies remain incapable of providing a comprehensive treatment that can prevent restenosis and inflammation while concurrently preserving the endothelial layer, which is vital for both vascular healing and preventing thrombosis and neoatherosclerosis. Due to the non-selective mechanism of action of the drugs eluted from the DES, ST will remain a persistent risk, and measures must be implemented to minimize this risk. For example, ensuring that patients are both able and likely to comply with at least 12 months of dual anti-platelet therapy before DES implantation together with ensuring optimal stent deployment during PCI are two effective ways of reducing this incessant risk of ST [41]. 

To date, cell-selective drugs that can discriminate between proliferating VSMCs, inflammatory cells and ECs are not available. Considering that vascular ECs provide crucial protection against thrombosis, lipid uptake, and inflammation, it is of paramount importance to develop a cell-selective therapy that can inhibit VSMC proliferation and inhibit inflammatory cell infiltration, yet spare ECs to carry on their vital functions. 

**Figure 2:**
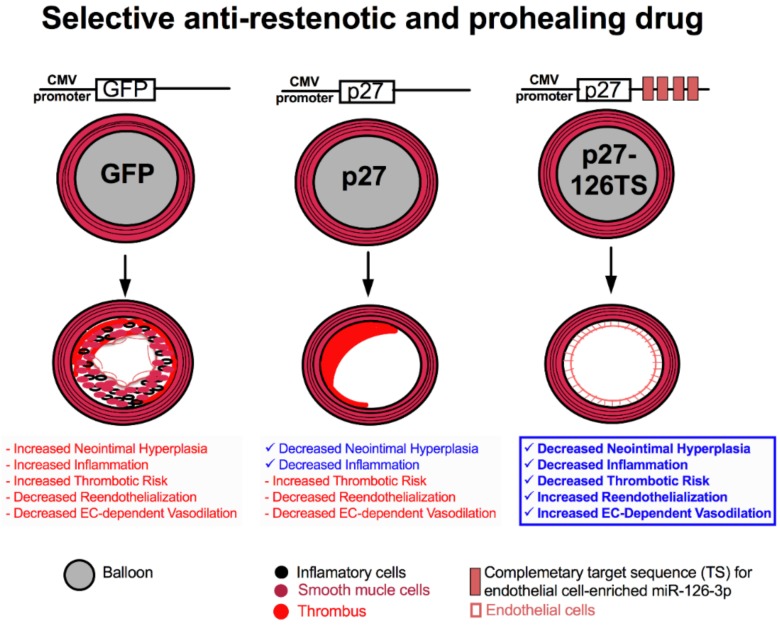
Graphic schematic depicting the cell-selective therapy capable of preventing restenosis and allowing for reendothelialization. Balloon injury and control green fluorescent protein (GFP) treated rat coronary artery results in neotintimal hyperplasia, infiltration of inflammatory cells, and an increased risk of thrombosis, accompanied by a severe reduction in reendothelialization and loss of endothelial cell-dependent vasodilation. Balloon injury and non-cell selective p27 treated rat coronary artery decreased neointimal hyperplasia and inflammation, but increased thrombosis due to decreased reendothelialization endothelial cell-dependent vasodilation. However, balloon injury and treatment with the cell-selective p27-126TS decreased neotintimal hyperplasia and inflammation, but also allowed for complete reendothelialization and effective endothelial cell-dependent vasodilation and greatly diminished the risk of thrombosis. EC: endothelial cell; CMV: Cytomegalovirus.

In response to this challenge, we have developed an innovative “microRNA (miRNA)-based cell-selective therapy” to selectively target VSMC and inflammatory cells while protecting ECs, thereby enabling them to reendothelialize vessel walls and maintain their crucial function (Figure 2) [42]. 

**Figure 3:**
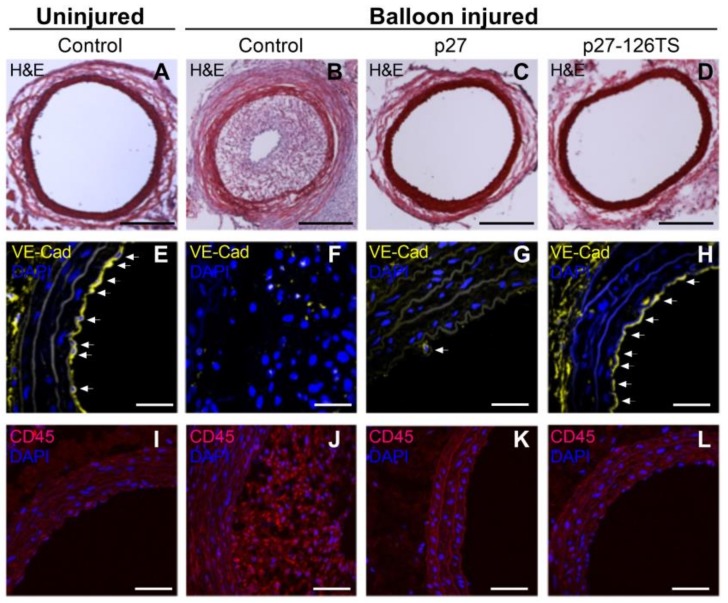
Treatment with p27-126TS prevents restenosis and allows for complete reendothelialization of the vascular wall. (**A**–**D**). Representative images of uninjured (**A**), or balloon injured rat carotid arteries treated with GFP (**B**), p27 (**C**), or p27-126TS (**D**) followed by hematoxylin and eosin (H&E) stain. Original magnification 10×, scale bars represent 500 μM. (**E**–**H**) Representative images of uninjured (E), or balloon injured rat carotid arteries treated with GFP (**F**), p27 (**G**), or p27-126TS (**H**) immunostained for vascular endothelial cell marker vascular endothelial-cadherin (VE-Cadherin). White arrows indicate VE-Cadherin positive endothelial cells. (**I**–**L**) Representative images of uninjured (**I**), or balloon injured rat carotid arteries treated with GFP (**J**), p27 (**K**), or p27-126TS (**L**) immunostained for the pan-inflammatory marker CD45. (**E**–**L**) Nuclei were counterstained with DAPI. Original magnification 60×, scale bars represent 100 μM.

miRNAs are a class of endogenous small noncoding RNAs that use base pairing to direct RNA-induced silencing complexes to specific mRNA transcripts containing partially or fully complementary sequences, resulting in the degradation or translational inhibition of the target mRNA [43,44]. The EC-specific miR-126 is a pivotal regulator of vascular integrity and angiogenesis, and is upregulated in arterial injury sites and atherosclerotic plaques [45,46,47]. We took advantage of this EC-specific miR-126 to design a unique adenoviral vector, encoding the CDK inhibitor p27 and incorporated four complementary target sequences for the mature miR-126-3p strand at its 3′ end (p27-126TS) (Figure 2) [42]. Testing the efficacy of this approach in a rat carotid balloon injury model revealed that local overexpression of p27 following balloon injury resulted in complete inhibition of neointimal formation and inflammation, but did not allow for reendothelialization of the vessel. These results are remarkably similar to those observed in vessels implanted with a DES that elutes currently used non-specific drugs. On the other hand, p27-126 treated vessels after balloon injury inhibited vascular neointimal formation and inflammation but also allowed for complete reendothelialization of the vessel (Figure 3). This preclinical study also illustrated that p27-126 treated vessels reduced hypercoagulability and restored the EC-mediated vasodilatory response to acetylcholine [42].

Building on these results, we employed the same miRNA-based cell-selective strategy in a hypercholesterolemic atherogenic rabbit model. Following balloon injury, local treatment of the carotid arteries with p27-126TS significantly reduced the neointimal area and accumulation of neointimal macrophages, and greatly improved reendothelialization. These results indicate the feasible use of this single treatment that combines the antirestenotic therapy with enhanced reendothelialization, even in the presence of hypercholesterolemic atherogenic conditions. This approach provides the base for designing the next generation of cell-selective targeting in PCI aimed at reducing the need for prolonged dual anti-platelet drug regimens.

### 6.2. Precision Medicine and Coronary Artery Disease

In parallel to the technological advances in the stent design, individualized treatment that takes into consideration the individual’s genetic lifestyle and environment to tailor healthcare decisions and treatments to the individual patient are required.

The Human Genome Project laid the groundwork for the development of precision medicine, also known as personalized medicine. In recent years, many preclinical and clinical trials have rapidly expanded the field of precision medicine in CAD. Genome-wide association studies (GWAS) have identified a multitude of single nucleotide polymorphisms (SNPs) that increase the susceptibility to develop CAD and in-stent restenosis [48], while others were associated with response to antiplatelet therapy [49]. Therefore, genetic testing in the clinical setting will help physicians when making medical decisions to improve the outcomes associated with PCI and lower healthcare cost [50].
